# Microglial activation is raised in preclinical Alzheimer’s disease and associated with covert memory impairment

**DOI:** 10.3389/frdem.2025.1745571

**Published:** 2026-01-27

**Authors:** Pernille Louise Kjeldsen, Lasse Stensvig Madsen, Peter Parbo, Rola Ismail, Joel Fredrik Astrup Aanerud, Malene Kaasing, Malene Flensborg Damholdt, Simon Fristed Eskildsen, Leif Østergaard, David James Brooks

**Affiliations:** 1 Department of Clinical Medicine, Aarhus University, Aarhus, Denmark; 2 Department of Nuclear Medicine and PET, Aarhus University Hospital, Aarhus, Denmark; 3 Centre of Functionally Integrative Neuroscience, Aarhus University, Aarhus, Denmark; 4 Department of Nuclear Medicine, Odense University Hospital, Odense, Denmark; 5 Department of Nuclear Medicine, Vejle Hospital, Vejle, Denmark; 6 Department of Psychology, Aarhus University, Aarhus, Denmark; 7 Translational and Clinical Research Institute, Newcastle University, Newcastle upon Tyne, United Kingdom

**Keywords:** Alzheimer’s disease, amyloid, cognition, memory, microglia, PET

## Abstract

**Background:**

Alzheimer’s disease (AD) is a continuum between normal health and dementia with a long preclinical phase, during which AD pathologies start to emerge, but where there are not yet any overt symptoms. The hallmark pathologies of AD are extracellular *β*-amyloid (Aβ) plaques and intra-neuronal neurofibrillary tangles (NFTs). Aβ deposition is present at the preclinical stage. Additionally, raised microglial activation is a key factor in AD. However, its exact timing and role is still unclear. This exploratory study investigated the prevalence of microglial activation and its association with Aβ deposition and memory impairment in preclinical AD.

**Methods:**

A total of 19 preclinical AD subjects with no cognitive complaints but abnormal Aβ deposition present on ^11^C-Pittsburgh Compound B (^11^C-PiB PET) and 10 healthy subjects with no cognitive complains or abnormal Aβ deposition on ^11^C-PiB PET underwent ^11^C-PK11195 PET (^11^C-PK). Additionally, the preclinical AD subjects underwent formal cognitive testing with sensitive memory tests, including the Rey Auditory Verbal Learning Test, the Rey Complex Figure Test, and the Face-Name Associative Memory Exam.

**Results:**

Microglial activation was raised in occipital and parietal cortices in preclinical AD subjects compared to healthy controls (*p* < 0.01). In the preclinical subjects there were significant positive correlations between Aβ load and microglial activation in parietal areas (*p* < 0.01). Finally, in the preclinical subjects, there were significant negative correlations between microglial activation and memory test performance in selected cortical areas (*p* < 0.01).

**Conclusion:**

Microglial activation was significantly raised in preclinical AD cases with no cognitive complaints and associated with impaired memory test performance. This suggests that microglial activation is present before overt clinical symptoms emerge and may be detrimental to cognition even at this early stage.

## 1 Introduction

The pathophysiological processes leading to Alzheimer’s disease (AD) represent a biological continuum with disease changes emerging in the brain over a decade prior to clinical symptoms (Sperling et al., 2011). AD pathology comprises extracellular deposits (plaques) of *β*-amyloid (Aβ) protein and intracellular neurofibrillary tangles of paired helical tau filaments (NFTs) (Braak and Braak, 1991), accompanied by microglial activation (Combs, 2009), and neurodegeneration. Of these, Aβ aggregation is believed to occur first (Pike et al., 2007; Aizenstein et al., 2008; Rentz et al., 2010). Given the view that these pathologies show in the brain years before the onset of clinical symptoms, there has been a need to better characterise the early, preclinical stage of the disease. To this end, the National Institute on Aging – Alzheimer’s Disease workgroups defined ‘preclinical AD’ as older individuals with incidental brain Aβ deposition, but no overt cognitive impairment (Sperling et al., 2011).

There is an extensive literature describing the microglial-mediated inflammatory response in AD. Previous studies have reported the presence of microglial activation in preclinical AD stages (Brosseron et al., 2014; Nordengren et al., 2019; Rauchmann et al., 2022; Wang et al., 2022; Zeng et al., 2022). In AD brains, activated microglia are closely associated with both Aβ plaques and tau tangles. Aβ plaques and NFT accumulation both trigger microglial activation but, conversely, activated microglia also triggers Aβ aggregation (Fruhwürth et al., 2024) and NFT formation (Dani et al., 2018). The exact role of microglial activation in AD, however, is still unclear. It has been suggested that microglial activation may occur in two phases over the course of AD (Fan et al., 2017; Pereira et al., 2022). In early stages of AD, microglial activation may be protective, clearing Aβ fibrils, whereas in later stages, as Aβ clearance fails, cortical NFTs aggregate, and neuronal loss occurs, microglial activation becomes toxic with the promotion of activated astrocytes and release of inflammatory cytokines (Barger and Harmon, 1997). Microglia may be particularly sensitive in the ageing brain due to age-related weakening caused by oxidative stress, mitochondrial dysfunction, and other cellular and synaptic changes (Malvaso et al., 2023). However, whether microglial activation is protective in some AD stages or is always harmful in AD is still debated (Rodriguez-Vieitez et al., 2024; Roveta et al., 2024; Houlihan et al., 2025; Yeh et al., 2025).

In AD, the most common cognitive symptom is that of memory impairment (Vyhnálek et al., 2019). Aβ and NFT load have been extensively studied in relation to memory impairment and, at different disease stages, both Aβ load and NFT burden can correlate with the degree of memory impairment (Hanseeuw et al., 2019). The exact effect of microglial activation on memory impairment is less clear, although there is evidence that in prodromal disease, microglial activation also correlates with memory impairment (Kreisl et al., 2013; Yeh et al., 2025), and is even predictive of memory decline (Wang et al., 2022).

The isoquinoline PET ligand, ^11^C-(R)-PK11195 (^11^C-PK), can be used to visualise 18 kDa translocator protein (TSPO) expression *in vivo* with PET imaging. The mitochondria of activated microglia express TSPO on their outer membrane, and ^11^C-PK PET can bind to an isoquinoline binding site on TSPO, thus detecting TSPO expression (Cagnin et al., 2001). TSPO expression is not necessarily unique to activated microglia, as some resting microglia and some astroglia may also express it, albeit to a lesser degree than activated microglia (Wijesinghe et al., 2025). Previous imaging studies have shown microglial activation with raised TSPO binding in early clinical AD, mild cognitive impairment due to AD (AD-MCI), and healthy individuals who later progressed to AD-MCI (Okello et al., 2009; Kreisl et al., 2013; Parbo et al., 2017; Ismail et al., 2020; Wang et al., 2022). To date, however, only a few studies have used TSPO PET to examine microglial activation in preclinical AD, and none have comprehensively examined its relationship with both amyloid and subtle memory changes.

The aim of this exploratory study was to assess the prevalence of microglial activation using ^11^C-PK PET in preclinical AD subjects, here defined as subjects with cortical Aβ plaques on ^11^C-PiB PET but no overt cognitive decline, and to evaluate its association with Aβ load and memory performance.

## 2 Methods

### 2.1 Subjects

#### 2.1.1 Preclinical AD subjects

A total of 64 subjects (age range: 55–75) underwent cognitive screening with the Mini-Mental State Examination (MMSE) and ^11^C-PiB PET. Of these, 19 subjects were found to have abnormal Aβ deposition on ^11^C-PiB PET and subsequently underwent ^11^C-PK PET.

The inclusion criteria were: (i) age between 50 and 80 at the time of inclusion, (ii) abnormal Aβ deposition present on a ^11^C-Pittsburgh Compound B (^11^C-PiB) PET scan.

The exclusion criteria were: (i) cognitive decline as indicated by a MMSE score < 26 (Folstein et al., 1975), (ii) clinically relevant symptoms of depression as indicated by a 15-item Geriatric Depression Scale (GDS-15) score > 5 (Yesavage et al., 1982), (iii) significant systemic, neurological, or psychiatric disorders, and (iv) contraindications to MRI.

#### 2.1.2 Control subjects

Further, an additional 10 subjects (age range: 65–80) from a previous study (Parbo et al., 2017; Ismail et al., 2020) were retrospectively included as controls. The previous study was carried out by the same research group, and thus, the recruitment strategies, inclusion and exclusion criteria, and scanning protocols for the healthy subjects were the same as for the preclinical AD subjects. These subjects were all overtly healthy, were cognitively intact on the MMSE, did not have any memory complaints, and were not found to have abnormal Aβ deposition on ^11^C-PiB PET.

### 2.2 Magnetic resonance imaging (MRI)

High-resolution 3D MRI of the brain was performed with a 3 T MRI scanner (Skyra Magnetom, *Siemens, Erlangen, Germany*) as previously described (Parbo et al., 2017; Madsen et al., 2023). Volumetric T1-weighted and fluid-attenuated inversion recovery (FLAIR) T2-weighted MRI images were obtained. An experienced neuroradiologist visually inspected the images for abnormalities to ensure that the subjects did not have any other underlying neurological disease.

### 2.3 Positron emission tomography (PET)

^11^C-PiB and ^11^C-PK PET were performed with a High-Resolution Research Tomograph (HRRT, CTI/Siemens, Knoxville, TN) scanner as previously described (Parbo et al., 2017). An experienced nuclear medicine specialist visually inspected the images to identify subjects with abnormal Aβ deposition.

#### 2.3.1 Amyloid PET

We used ^11^C-PiB PET to measure levels of fibrillar Aβ in the brain. A mean dose of 394 (+/− 39) MBq ^11^C-PiB was injected through the subject’s intravenous catheter followed by a 10 mL saline flush. The subject rested outside the scanner for 30 min before lying supine in the scanner. The total scan time was 50 min. List-mode PET was acquired 40–90 min post injection and then rebinned into 5 × 10 min. Time frames.

#### 2.3.2 TSPO pet

We used ^11^C-PK PET to measure TSPO expression of activated microglia levels in the brain. A mean dose of 398 (+/− 35) MBq ^11^C-PK was injected through the subject’s intravenous catheter followed by a 10 mL saline flush after a 30 s. background frame prior to injection. The subject lay supine in the scanner prior to injection. The total dynamic scan time in list mode was 60.5 min. The frames were subsequently rebinned as a 1 × 30 s. background frame and 6 × 10 s., 2 × 30 s., 2 × 60 s., 3 × 120 s., and 10 × 300 s time frames.

### 2.4 Neuropsychological assessment

The preclinical AD subjects were all assessed with an extensive neuropsychological test battery, including a range of sensitive memory tests. The selected sensitive memory tests included the Rey Auditory Verbal Learning Test (RAVLT) (Rey, 1964; Schmidt, 1996), which is a verbal learning test, in which the subject is presented with a word list five times, asked to immediately recall it and, after a delay, is asked to recall it again, the Rey Complex Figure Test (RCFT) (Rey, 1941; Meyers and Meyers, 1995), in which the subject is presented with a complex figure, asked to draw it, and after a delay asked to redraw it from memory, and the Face Name Associative Memory Test (FNAME) (Rentz et al., 2011), in which the subject is presented with a number of associated faces, names, and occupations, asked to immediately recall them, and after a delay asked to recall them again. All the preclinical subjects completed the tests, except for one who did not complete the RCFT. The healthy controls underwent a less extensive neuropsychological test battery, including some, but not all, of the tests from the more extensive test battery. The tests batteries can be found in the Supplementary Table 1.

### 2.5 Image analysis

MINC software^1^ was used for image analysis. MRI scans were segmented into grey matter (GM), white matter (WM), and cerebrospinal fluid (CSF) images. The individual PET images were co-registered to the corresponding MRI images and spatially normalised into MNI space.

The spatially normalised ^11^C-PiB images were summed from 60–90 min. and the activity in each voxel was divided by the mean activity from the individual subjects’ cerebellar GM to generate ^11^C-PiB standardized uptake value ratio (SUVR) images.

Parametric maps of ^11^C-PK binding potential (BP_ND_) were generated at a voxel level using the simplified reference tissue model (SRTM) (Lammertsma and Hume, 1996) implemented in MATLAB. As ^11^C-PK PET signals are subtle and spatially diffuse, a Supervised Cluster Analysis with 6 classes (SVC6) (Turkheimer et al., 2007) was used to localise clusters of voxels from the dynamic images of each individual subject that either showed the kinetics of specific tissue binding or provided a reference tissue input function representative of normal GM uptake kinetics (Ismail et al., 2020), thus focusing attention on regions with meaningful microglial activation.

A probabilistic atlas (Hammers et al., 2003) was used to define regions of interest (ROIs) within cortical GM. A volume-weighted average of frontal, lateral and posterior temporal, precuneus, parietal, and posterior cingulate cortical regions was used to form a composite ROI for both ^11^C-PiB and ^11^C-PK (Parbo et al., 2017). The individual mean of ^11^C-PiB SUVR and ^11^C-PK BP_ND_ images was calculated within the composite ROI, using the unsmoothed images to minimise spill-in/spill-out contamination. Subsequently, ^11^C-PiB SUVR images were smoothed with a 6 mm Gaussian kernel.

### 2.6 Cortical surface statistical mapping

Cortical surface based statistical mapping was used to localise significant correlations between ^11^C-PiB and ^11^C-PK uptake as well as between ^11^C-PK uptake and memory test scores. One of the 19 subjects had an incomplete MRI that did not allow cortical statistical surface maps to be computed. Hence, surface statistical maps were generated for 18 subjects.

The cortical surfaces were generated with FACE (Fast Accurate Cortex Extraction) using a previously published protocol (Ismail et al., 2020). FACE is used to show correlations in cortical regions of interest, as it iteratively fits topologically correct surface meshes to the WM-GM and the GM-CSF interfaces with sub-voxel precision using pre-processed T1-weighted MRI images (Eskildsen et al., 2005; Eskildsen and Østergaard, 2006). Specifically, the middle cortical layer was estimated as the surface between the grey matter–cerebrospinal fluid interface and the white matter–grey matter interface. The parametric images were mapped onto their corresponding cortical surfaces. The cortical surfaces were then moved to parametric native space using a rigid body co-registration. The sampling was performed in parametric native space. Individual cortical surfaces were then moved on to a common template space using a non-linear co-registration. Finally, the final parametric surfaces were smoothed with a 20 mm FWHM geodesic Gaussian kernel along the cortex.

^11^C-PiB SUVR and ^11^C-PK BP_ND_ images were mapped to a mid-cortical surface and subsequentially smoothed using a 20 mm geodesic Gaussian kernel. This enabled isolated smoothing of the GM signal and smoothing of functionally similar regions along the cortex. Prior to the cortical surface mapping, the ^11^C-PiB SUVR and ^11^C-PK BP_ND_ images were smoothed with a 3x3x3 mm FWHM Gaussian filter to compensate for the lower PET resolution and potential inaccuracies in the co-registration.

Correlations between tracer uptake and cognitive scores were calculated at each point on the cortical surface using a general linear regression model implemented in Python 3.7.3 (Python Software Foundation). All statistical correlation maps were thresholded with *p* < 0.01 and family-wise error rate (FWER) corrected at *p* < 0.05 using cluster-extent based thresholding.

Visbrain software (Combrisson et al., 2019) was used to visualize the cortical surface maps. The findings are presented as cortical areas of FWER corrected significant correlation.

### 2.7 Statistics

Non-imaging data were statistically interrogated using IBM Statistical Packages for Social Sciences (SPSS) version 28.0. Descriptive statistics were used to examine demographic variables. The correlations between mean ^11^C-PiB SUVR and ^11^C-PK BP_ND_ were computed using linear regressions. Correlations between mean ^11^C-PK BP_ND_ and cognitive test scores were computed using the non-parametric Spearman’s rank correlation due to cognitive test scores typically being skewed in small samples. All analyses were corrected for age. A *p* < 0.05 was considered statistically significant. The exact *p*-value is given where possible and otherwise reported as *p* < 0.05, *p* < 0.01, or *p* < 0.001.

## 3 Results

### 3.1 Sample characteristics

Demographics for the subjects are summarised in Table 1.

**Table 1:** Demographics for all participants.

Demographics	Healthy subjects (*n* = 10) Mean (SD)	Preclinical AD subjects (*n* = 19) Mean (SD)
Age	67.9 (7.2; range: 68–80 years)	70.0 (3.0; range: 63–75 years)
Gender, % women	33.3% (*n* = 3)	36.8% (*n* = 7)
MMSE^†^	28.3 (1.70)	28.8 (0.85)
GDS-15^‡^	0.40 (0.7)	0.89 (1.2)
Fazeka’s score	1.1 ± 0.9	1.52 ± 0.6

^†^ MMSE = Mini-Mental State Examination; ^‡^ GDS-15 = 15-item Geriatric Depression Scale.

### 3.2 Microglial activation

**Figure 1:**
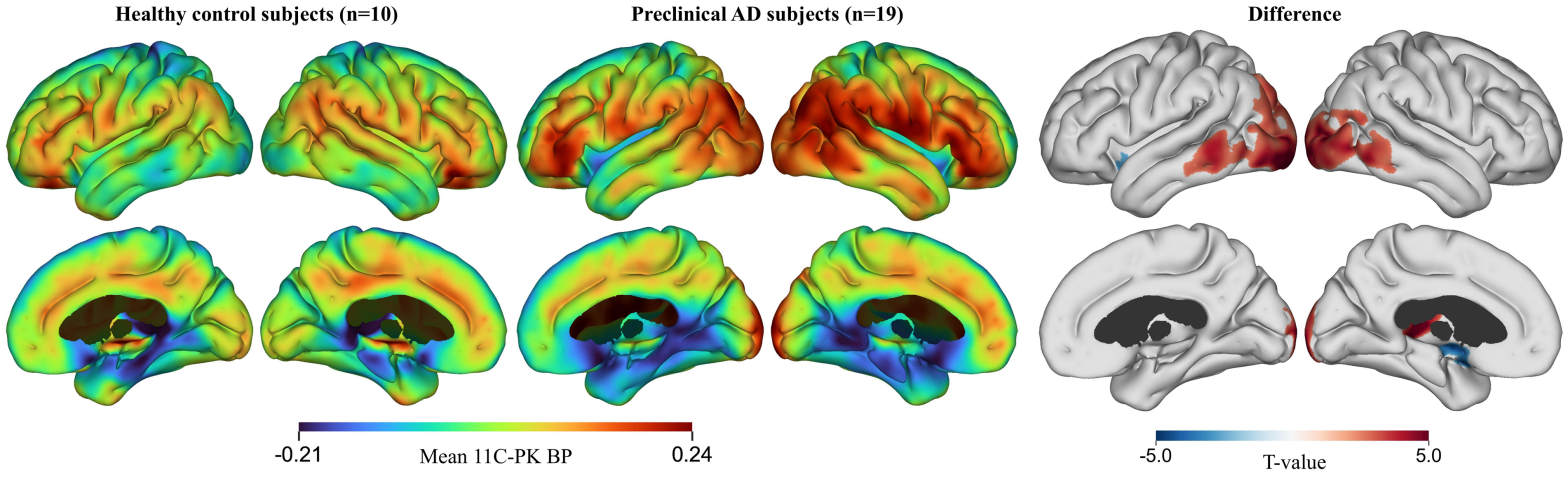
Cortical surface maps of ^11^C-PK binding in healthy controls and preclinical AD subjects corrected for age and sex. FWE-correction at *p* < 0.01.

A cortical statistical analysis revealed significant differences in ^11^C-PK binding in the occipital and temporal cortices as well as the thalamus between the healthy controls and the preclinical AD subjects (*p* < 0.01) after adjusting for age, with the preclinical AD subjects showing higher ^11^C-PK binding overall (Figure 1; Table 2).

**Table 2:** Cortical regions with significant differences in ^11^C-PK binding between healthy controls and preclinical AD subjects corrected for age and sex. FWE-correction at *p* < 0.01.

	Anatomical location	Hemisphere	Cluster area (mm2)	MNI coordinates (x, y, z)	Cluster FWE *p*-value
1	Inferior occipital gyrus	Left	3,779	[−29, −98, −10]	2.6E-06
2	Calcarine sulcus	Right	2,689	[16, −104, −2]	3E-06
3	Thalamus	Left	255	[−3, −27, 7]	0.025

### 3.3 Correlations between amyloid and microglial activation

**Figure 2:**
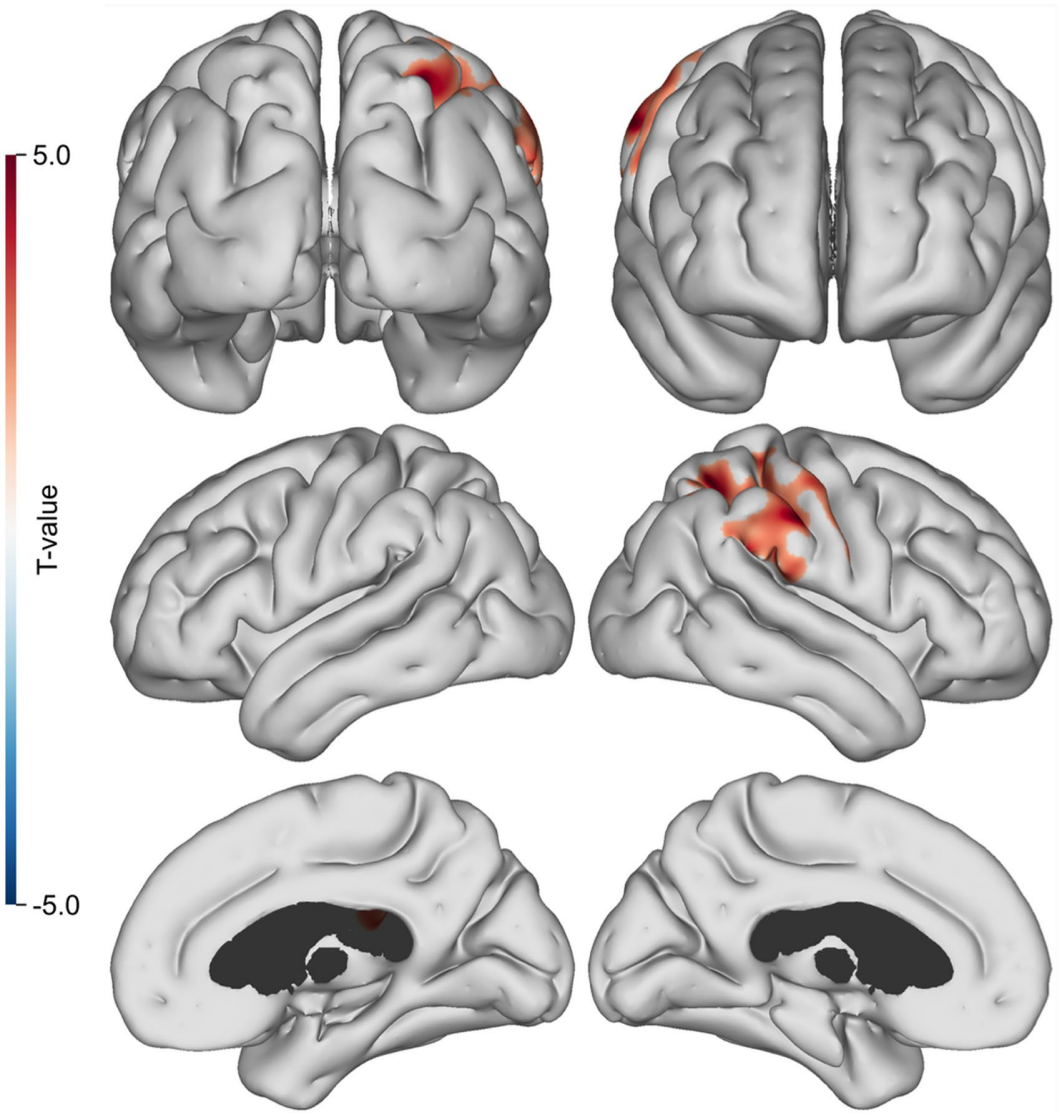
Cortical surface map of Aβ and TSPO correlations. Highlighted regions indicate where ^11^C-PiB and ^11^C-PK are positively correlated (*n* = 19, cluster FWER, *p* < 0.01).

In the preclinical AD subjects, cortical statistical surface map analysis revealed statistically significant positive correlations between ^11^C-PiB and ^11^C-PK uptake in the right parietal lobe after adjusting for age (*p* < 0.01) (Figure 2).

### 3.4 Correlations between microglial activation and cognitive scores

The preclinical AD subjects’ memory test scores are described in Table 3.

**Table 3:** Neuropsychological test performance for all preclinical AD subjects.

Neuropsychological test	Subjects (*n* = 19) Mean (SD)
RAVLT^†^ − Total immediate recall	45.26 (8.27)
RAVLT^†^ − Delayed recall	8.21 (3.72)
RAVLT^†^ − Recognition	13.11 (2.08)
FNAME^‡^ − Immediate recall	4.11 (2.278)
FNAME^‡^ − Delayed recall	6.53 (3.55)
FNAME^‡^ − Total	45.32 (16.02)
RCFT^§^ – Copy	26.47 (6.05) (^¶^*n* = 18)
RCFT^§^ – Delayed recall	12.61 (6.92) (^¶^*n* = 18)
RCFT^§^ – Recognition	19.94 (1.47) (^¶^*n* = 18)

^†^ RAVLT = Rey Auditory Verbal Learning Test; ^‡^ FNAME = Face-Name Associative Memory Exam; ^§^ RCFT = Rey Complex Figure Test. ^¶^ One less participant due to testing error.

In the preclinical AD subjects, partial correlation analysis with age as a covariate using FDR correction for multiple comparisons revealed a statistically significant inverse correlation between ^11^C-PK binding and the Rey Auditory Verbal Learning Test (RAVLT) – Delayed Recall scores (*r* = −0.492, *p* = 0.038) as well as between ^11^C-PK uptake and the Face-Name Associative Memory Test (FNAME) – Delayed Recall scores (*r* = −0.558, *p* = 0.016). There was no statistically significant correlation between ^11^C-PK uptake and the Rey Complex Figure Test (RCFT) scores (*r* = −0.312, *p* = 0.201).

**Figure 3:**
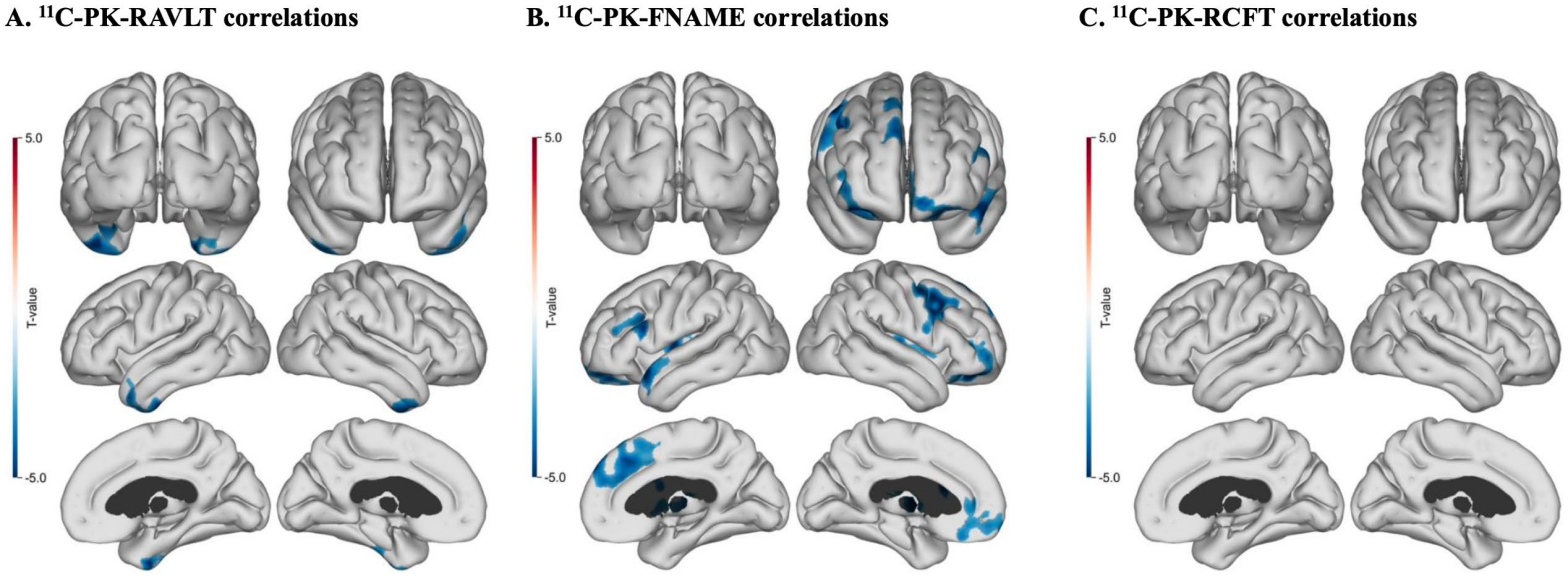
Cortical surface maps of ^11^C-PK uptake and memory test correlations for all subjects with age added as a co-variate. **(A)** Significant inverse correlations between ^11^C-PK uptake and RAVLT – delayed recall scores (*n* = 19, cluster FWE rate, *p* < 0.01). **(B)** Significant inverse correlations between ^11^C-PK uptake and FNAME – delayed recall scores (*n* = 19, luster FWE rate, *p* < 0.01). **(C)** One significant regional correlation between ^11^C-PK uptake and RCFT – delayed recall scores (*n* = 18, luster FWE rate, *p* < 0.01).

Cortical surface analyses with age as a covariate revealed statistically significant inverse correlations between ^11^C-PK uptake and RAVLT – Delayed Recall scores (Figure 3; Table 4) in the right inferior temporal gyrus and in the right fusiform gyrus (*p* < 0.01) (Figure 3A) as well as between ^11^C-PK uptake and FNAME – Delayed Recall subtest scores for name recall bilaterally in frontal cortices, bilaterally in fusiform gyrus as well as in the left superior temporal pole, the right anterior cingulate gyrus, and in the Rolandic operculum (*p* < 0.01) (Figure 3B). There was one cluster of significant correlation between ^11^C-PK uptake and RCFT – Delayed Recall test scores in the left parahippocampal gyrus (*p* < 0.01) (Figure 3C).

**Table 4:** Cortical surface map correlations between ^11^C-PK PET uptake and memory test scores.

Neuropsychological test	Cluster area (mm^2^)	MNI coordinates (x, y, z)	Anatomical location	FWER corrected cluster *p*-value
RAVLT^1^ – Delayed Recall	462	[−44, −34, −27]	Inferior temporal gyrus left	0.017
RAVLT^1^ – Delayed Recall	496	[25, −4, −45]	Fusiform gyrus right	0.0032
FNAME^2^ – Delayed Recall	1,120	[−11, 64, −19]	Superior orbitofrontal left	0.00014
FNAME^2^ – Delayed Recall	760	[−38, 21, −17]	Inferior orbitofrontal left	0.0014
FNAME^2^ – Delayed Recall	1,004	[25, 18, −25]	Inferior orbitofrontal right	0.00026
FNAME^2^ – Delayed Recall	646	[42, 27, 42]	Middle frontal gyrus right	0.0017
FNAME^2^ – Delayed Recall	1,000	[−4, 58, 22]	Medial superior frontal gyrus left	0.0044
FNAME^2^ – Delayed Recall	1,655	[4, 53, 21]	Medial superior frontal gyrus right	<0.0001
FNAME^2^ – Delayed Recall	475	[−53, 15, −21]	Superior temporal pole left	0.0012
FNAME^2^ – Delayed Recall	938	[−46, −51, −19]	Fusiform gyrus left	0.0013
FNAME^2^ – Delayed Recall	402	[45, −39, −26]	Fusiform gyrus right	0.025
FNAME^2^ – Delayed Recall	695	[3, 14, 26]	Anterior cingulate gyrus right	0.027
FNAME^2^ – Delayed Recall	379	[48, −13, 14]	Rolandic operculum right	0.018
RCFT^3^ – Delayed Recall	354	[−18, −10, −24]	Parahippocampal gyrus left	0.042

1. RAVLT = Rey Auditory Verbal Learning Test, 2. FNAME = Face-Name Associative Memory Exam, and 3. RCFT = Rey Complex Figure Test.

## 4 Discussion

The goal of this exploratory study was to investigate the prevalence of raised microglial activation as well as its relationships with β-amyloid load and memory test performance in preclinical AD. We found that microglial activation was raised targeting the occipital cortex and the posterior temporal cortex in preclinical AD subjects compared to healthy controls. We also found that, when present, the spatial distribution of microglial activation tracked that of Aβ deposition in the parietal cortex in the preclinical AD subjects. Further, we found significant inverse correlations between levels of microglial activation and test performance on two sensitive memory tests, RAVLT and FNAME, in frontal and temporal cortices in the preclinical subjects.

Previous studies using TSPO PET have shown that microglial activation can be detected in the majority of MCI due to AD cases (Parbo et al., 2017, Ismail et al., 2020) as well as in subjects with early clinical AD (Tournier et al., 2020; Wang et al., 2022). Our study shows that microglial activation can already be detected using TSPO PET in subjects with preclinical AD. However, it is worth noting in this regard that ^11^C-PK PET has some important methodological limitations. Although, TSPO upregulation as measured with ^11^C-PK PET has often been interpreted as a marker of activated microglia, it has been shown that TSPO is also expressed by some resting microglia and astrocytes, and all endothelial cells (Chauveau et al., 2008; Ghadery et al., 2019; Chauveau et al., 2025). Thus, while TSPO upregulation may be a result of microglial activation (Wijesinghe et al., 2025), it may also result from other factors. Additionally, ^11^C-PK PET cannot distinguish between different microglia phenotypes. There are three overarching microglia phenotypes, including resting microglia, active anti-inflammatory microglia, and active pro-inflammatory microglia (Wei and Li, 2022; Wendimu and Hooks, 2022). As all of them may express TSPO, ^11^C-PK PET cannot be used to discriminate them. Additionally, a distinct class of microglia named disease-associated microglia (DAM) has been discovered to occur in neurodegenerative diseases such as AD in response to brain pathology (Keren-Shaul et al., 2017). DAMs emerge in brain regions with neurodegenerative pathology and are directly involved in clearing toxic proteins in these regions. DAMs are not purely harmful or purely protective. They may serve a more protective role early in the disease process and become more harmful one later on, however, their roles are still not fully known, especially in humans (Fujikawa and Tsuda, 2023; Malvaso et al., 2023). As with the overarching phenotypes, ^11^C-PK PET cannot reveal whether microglia in a certain region are DAMs or what the cells are doing at a specific time. Further, ^11^C-PK PET may also be a measure of microglial density in humans more than levels of microglial activation (Nutma et al., 2023). In general, the role of microglia in AD is very complex, and at the time, there are no microglia PET tracers that can shed light *in vivo* on all of these complexities.

While all our preclinical AD subjects had abnormal A*β* deposition, not all of them had raised microglial activation, lending support to the argument that Aβ plaques occur ahead of microglial activation in preclinical AD. However, it is also possible that ^11^C-PK PET is not sensitive enough to detect as low levels of microglial activation that may be present in preclinical AD. To fully explore the temporal relationship between Aβ and microglial activation, a longitudinal study with serial Aβ and microglia PET on a larger cohort of preclinical subjects without abnormal Aβ deposition at entry would be required.

Microglial activation has consistently been associated with Aβ plaques in AD at post-mortem and *in vivo* studies. In our study, we were able to show significant correlations between overall cortical Aβ load and microglial activation in the parietal cortex. Although, we found other correlations between cortical Aβ load and microglial activation in our subjects (Supplementary Table 1), only those in the parietal cortex remained significant after FWE correction. It has been observed that β-amyloid deposition initially rises steadily and then plateaus. It is only possible to observe correlations between regional Aβ load and TSPO signals during the rising phase. The parietal region is one of the regions where the Aβ is continuing to rise in preclinical AD after Aβ deposition has begun, so we were able to find significant correlations between Aβ and microglial activation located here. However, the regional overlap between the two pathologies we detected was limited. As our sample size was small, we were likely also underpowered to detect the full extent of correlations between Aβ deposition and microglial activation.

The role of microglial activation along the AD continuum has been under debate for many years with some studies reporting that all microglial activation in AD is harmful to the brain, whereas others indicate that microglial activation, especially DAMs, may serve a protective role in the early stages of AD and only later take on a more toxic role as cortical tau deposition occurs (Amor et al., 2010). Our study is more in line with the former in that we found that microglial activation was associated with poorer performance on delayed memory tests, particularly associative memory test performance in our preclinical AD subjects. Previous studies (Yokokura et al., 2011; Kreisl et al., 2013; Wang et al., 2022) have shown an association between increasing microglial activation and memory impairment in preclinical AD, MCI due to AD and early AD, and our study is in line with these other findings. Another previous study (Madsen et al., 2024) also showed that raised microglial activation can act as a partial mediator of Aβ-related memory impairment in MCI due to AD. Unfortunately, our study was underpowered to examine whether this can also be the case in preclinical AD. Future studies with a larger cohort of preclinical AD subjects with and without abnormal Aβ deposition would be required to establish whether microglial activation is more harmful than protective in preclinical AD and its exact influence on memory. None of our preclinical AD subjects had tau PET, and thus, we cannot exclude that some of them may have had NFTs which could have affected their memory. However, given that our subjects did not have any cognitive complaints, it is unlikely that they would have had cortical NFTs. Aβ oligomers and microglia can both negatively impact memory on their own. Nonetheless, future studies should seek to incorporate tau PET along with Aβ and microglia PET.

Finally, microglial activation has been implicated in several other neurological diseases with neurodegenerative features (Pavese et al., 2006; Goulart et al., 2025; Ho, 2019). Uncovering the exact mechanisms of microglia in neurodegenerative diseases could aid in the development of new therapeutic targets in not just AD, but in other neurodegenerative diseases including, but not limited to, progressive multiple sclerosis as well (Malvaso et al., 2024).

### 4.1 Limitations and strengths

This study has limitations: The study was based on 19 subjects with preclinical AD, which is a small sample size. This is a cross-sectional study and we, therefore, do not know the longitudinal role that microglia will play in these subjects or their eventual outcome. We were only able to perform Aβ and TSPO PET, but not tau PET. Thus, we cannot rule out that some of our subjects had NFTs, and that it could have influenced the results. However, it is unlikely given that none had overt cognitive symptoms. ^11^C-PK PET has a high non-specific background signal, so we may have missed low levels of microglial activation. TSPO PET cannot distinguish between microglial inflammatory and protective phenotypes, including DAMs, and it may not only be a measure of microglial activation but rather microglial density (Nutma et al., 2023), making it difficult to know the exact implications of the signal.

However, the study also has strengths: Although the sample size is small, this is one of only a few studies that have performed ^11^C-PK PET and investigated, not only the presence of raised microglial activation, but also the associations between microglial activation and cognition in preclinical AD subjects with no cognitive complaints, and to show that raised microglial activation may be associated with subtle memory impairment.

Future studies with a larger cohort that can be studied both cross-sectionally and longitudinally are needed to better elucidate the roles of Aβ and microglial activation as well as tau on cognition along the preclinical and early AD continuum. We plan to perform such a study.

## 5 Conclusion

This exploratory study provides possible new insights into the presence, deposition, and effect of microglial activation in preclinical AD. We found that microglial activation is significantly raised in preclinical AD cases with abnormal Aβ deposition compared to healthy controls without Aβ, suggesting that microglial activation may present in AD before overt cognitive impairment emerges. Additionally, we found that raised microglial activation is negatively correlated with delayed memory test performance, thus suggesting a possible negative influence of microglia activation even in this early stage. Since our study was a small exploratory study, however, larger, longitudinal studies are needed to fully explore the extent to which microglial activation occurs and affects memory along the continuum of AD.

## Data Availability

The raw data supporting the conclusions of this article will be made available by the authors, without undue reservation.

## Footnotes

1
http://en.wikibooks.org/wiki/MINC
