# Supplementary material for: Microglial activation is raised in preclinical Alzheimer’s disease and associated with covert memory impairment
Source: Front Dement. 2026 Jan 27;4:1745571. doi: 10.3389/frdem.2025.1745571 (PMC12885982; doi:10.3389/frdem.2025.1745571)
Supplement: Supplementary file 1 [file Table_1.DOCX]

**Supplementary table 1. Full neuropsychological test batteries for both cohorts**

| **HEALTHY CONTROLS** | **PRECLINICAL AD** |
| --- | --- |
|  | |
| **Memory** | **Memory** |
| Rey Auditory Verbal Learning Test (RAVLT) | Rey Auditory Verbal Learning Test (RAVLT) |
| Logical Memory (WMS^1^) | Rey Complex Figure Test (RCFT) |
|  | Face-Name Associative Memory Test (FNAME) |
|  | |
| **Executive functions** | **Executive functions** |
| Trail Making Task A and B | Trail Making Task A and B |
| Digit Span Forwards and Backwards (WAIS^2^) | Digit Span Forwards, Backwards, and Ranked (WAIS^2^) |
| Golden Stroop | Golden Stroop |
| Coding |  |
|  | |
| **Visuo-spatial functions** | **Visuo-spatial functions** |
| Clock Drawing Task | Rey Complex Figure Test (RCFT) |
| Block Designs (WAIS^2^) | Block Designs (WAIS^2^) |
|  | Raven’s Progressive Matrices |
|  | |
| **Language** | **Language** |
| Boston Naming Test | Boston Naming Test |
| Verbal Fluency for Animals and S-words | Verbal Fluency for Animals and S-words |
|  | |
| **Abstraction** | **Abstraction** |
| Similarities (WAIS^2^) | Similarities (WAIS^2^) |
|  | Danish Adult Reading Test (DART) |
|  |  |

**Supplementary table 1. Full neuropsychological test batteries for both healthy control subjects and preclinical AD subjects.** Both test batteries were designed to evaluate a subject’s cognitive status. The test battery for the preclinical AD subjects was designed to be more focused so as to elucidate any subtle impairments in memory functions. 1) Wechsler’s Memory Scale, 2) Wechsler’s Adult Intelligence Scale.
